# A Novel Multi-Model High Spatial Resolution Method for Analysis of DCE MRI Data: Insights from Vestibular Schwannoma Responses to Antiangiogenic Therapy in Type II Neurofibromatosis

**DOI:** 10.3390/ph16091282

**Published:** 2023-09-11

**Authors:** Ka-Loh Li, Daniel Lewis, Xiaoping Zhu, David J. Coope, Ibrahim Djoukhadar, Andrew T. King, Timothy Cootes, Alan Jackson

**Affiliations:** 1 Division of Informatics, Imaging and Data Sciences, School of Health Sciences, Faculty of Biology, Medicine and Health, University of Manchester, Manchester M13 9PL, UK; ka-loh.li-2@manchester.ac.uk (K.-L.L.); timothy.f.cootes@manchester.ac.uk (T.C.); alan.jackson@manchester.ac.uk (A.J.); 2 Geoffrey Jefferson Brain Research Centre, University of Manchester, Manchester M13 9PL, UK; daniel.lewis-3@postgrad.manchester.ac.uk (D.L.); david.coope@manchester.ac.uk (D.J.C.); andrew.king@manchester.ac.uk (A.T.K.); 3 Department of Neurosurgery, Manchester Centre for Clinical Neurosciences, Salford Royal NHS Foundation Trust, Manchester Academic Health Science Centre, Manchester M13 9NT, UK; 4 Division of Neuroscience and Experimental Psychology, School of Biological Sciences, Faculty of Biology, Medicine and Health, University of Manchester, Manchester M13 9PL, UK; 5 Wolfson Molecular Imaging Centre, University of Manchester, 27 Palatine Road, Manchester M20 3LJ, UK; 6 Department of Neuroradiology, Manchester Centre for Clinical Neurosciences, Salford Royal NHS Foundation Trust, Manchester Academic Health Science Centre, Manchester M13 9NT, UK; ibrahim.djoukhadar@nca.nhs.uk; 7 Division of Cardiovascular Sciences, School of Medical Sciences, Faculty of Biology Medicine and Health, University of Manchester, Manchester M13 9PL, UK

**Keywords:** bevacizumab, DCE-MRI, neurofibromatosis type 2, prediction, treatment response

## Abstract

This study aimed to develop and evaluate a new DCE-MRI processing technique that combines LEGATOS, a dual-temporal resolution DCE-MRI technique, with multi-kinetic models. This technique enables high spatial resolution interrogation of flow and permeability effects, which is currently challenging to achieve. Twelve patients with neurofibromatosis type II-related vestibular schwannoma (20 tumours) undergoing bevacizumab therapy were imaged at 1.5 T both before and at 90 days following treatment. Using the new technique, whole-brain, high spatial resolution images of the contrast transfer coefficient (K^trans^), vascular fraction (v_p_), extravascular extracellular fraction (v_e_), capillary plasma flow (F_p_), and the capillary permeability-surface area product (PS) could be obtained, and their predictive value was examined. Of the five microvascular parameters derived using the new method, baseline PS exhibited the strongest correlation with the baseline tumour volume (*p* = 0.03). Baseline v_e_ showed the strongest correlation with the change in tumour volume, particularly the percentage tumour volume change at 90 days after treatment (*p* < 0.001), and PS demonstrated a larger reduction at 90 days after treatment (*p* = 0.0001) when compared to K^trans^ or F_p_ alone. Both the capillary permeability-surface area product (PS) and the extravascular extracellular fraction (v_e_) significantly differentiated the ‘responder’ and ‘non-responder’ tumour groups at 90 days (*p* < 0.05 and *p* < 0.001, respectively). These results highlight that this novel DCE-MRI analysis approach can be used to evaluate tumour microvascular changes during treatment and the need for future larger clinical studies investigating its role in predicting antiangiogenic therapy response.

## 1. Introduction

Dynamic contrast-enhanced MRI (DCE-MRI) is a medical imaging technique that can be used to assess tissue perfusion and permeability. It requires an injection of a gadolinium-based-contrast agent (GBCA) into the blood, whilst acquiring a series of dynamic MRI images as the contrast agent passes through the tissue of interest. By analysing the dynamic changes in GBCA concentration in the tissue, pharmacokinetic modelling can then be used to derive various parameters such as the volume of distribution, the rate of blood flow, and the permeability of the tissue, in turn, providing valuable information on drug delivery and efficacy. This technique is particularly useful for evaluating the effectiveness of anti-cancer therapies, as it can provide insight into how efficiently a drug is penetrating the tumour microenvironment and whether the tumour is responding to treatment [1,2,3,4,5,6,7,8].

Traditionally, a key limitation in DCE-MRI was achieving both simultaneous high spatial (HS) and high temporal (HT) resolution during data acquisition. High temporal resolution, especially during the arterial phase of the GBCA time-course, is critical if accurate quantification of kinetic parameters such as the fractional plasma volume (v_p_), transfer constant (K^trans^), and the fractional volume of extravascular extracellular space (v_e_) is to be achieved [9,10,11,12,13,14]. Usage of DCE-MRI as a clinical tool, however, also requires high spatial resolution and whole-brain coverage, especially when lesions are small, heterogenous, or widespread throughout the brain. A DCE-MRI technique that can provide both high spatiotemporal resolution and whole-brain coverage for quantitative microvascular analysis is therefore highly desirable [15,16,17], and in earlier works, it was demonstrated that a novel dual-temporal resolution (DTR) DCE-MRI-based data construction technique termed the LEGATOS (level and rescale the gadolinium contrast concentration curves of high temporal to high spatial) method could be used to provide this [18,19].

Previous studies employing DTR DCE-MRI and the LEGATOS technique have used the extended Tofts model (ETM) [18,19] to derive tissue-validated high spatial resolution estimates of K^trans^, v_p_, and v_e_ [20,21]. A key limitation of the ETM, however, is that the derived parameter K^trans^ is a hybrid parameter reflecting both capillary plasma flow (F_p_) and the capillary permeability-surface area product (PS) [22]. The ETM is also unable to accurately measure low-level permeability within normal-appearing brain tissue, which is of increasing importance in neuro-oncology and may help predict both prognosis and treatment response [23,24,25].

Recent studies have demonstrated that in addition to kinetic parameter estimation, absolute cerebral blood flow (CBF) estimates can also be derived from T1-weighted DCE-MRI through the use of the microsphere model and the maximum gradient approach [26,27]. In this technical proof of concept study, we demonstrate that incorporating ETM and the established ‘early time points’ method for absolute cerebral blood flow (ET-CBF) quantification [26] with the LEGATOS technique allows simultaneous evaluation of both tissue blood flow and permeability-based microvascular metrics [28]. In addition, we demonstrate that the integration of LEGATOS with the ETM and a previously described established hybrid first-pass Patlak plot (FP-PP) model [24] allows not only for high spatial resolution assessment of permeability effects within tumours but also high spatial resolution interrogation of low-level permeability effects within normal-appearing brain regions.

As a disease model to evaluate the presented multi-kinetic model technique, we retrospectively analysed previously acquired dual-injection DTR DCE-MRI data from a cohort of patients with neurofibromatosis type 2 (NF2) related vestibular schwannoma (VS) undergoing antiangiogenic (bevacizumab) therapy [4]. NF2 is a dominantly inherited tumour predisposition syndrome, the hallmark of which is the development of VS arising from the vestibulocochlear nerve bilaterally [29,30,31]. The management of these sometimes rapidly progressive VS can present a significant treatment challenge [32,33], and affected patients often develop, alongside bilateral VS, other tumours such as multiple meningiomas [34]. Trials of the anti-vascular endothelial growth factor (VEGF) antibody bevacizumab (Avastin©) in NF2 patients have demonstrated a reduction of VS growth rate [35] and regression in some cases [36], but there is variation in treatment results, with reported tumour response rates of 40–60% [35,36,37]. Previous studies have demonstrated that microvascular parameters derived from DCE-MRI and the ETM have possible treatment predictive value [4,35], and within this study, we sought to evaluate the applicability of the presented multi-model DCE-MRI analysis approach to this disease model. In particular, we sought to evaluate if this novel multi-model high spatial DCE-MRI analysis technique could be used to differentiate treatment-related changes in microvascular flow and permeability within the tumour microenvironment and identify parameters that may allow for better treatment response prediction in future clinical trials. 

### 1.1. Multi-Model LEGATOS Analysis Theory

#### 1.1.1. Construction of a 4D High Spatiotemporal Resolution GBCA Concentration-Volume Using the LEGATOS Method

The use of DTR DCE-MRI and the LEGATOS method for deriving whole-brain, high spatial resolution microvascular parameters have been previously described [20,21]. Low-dose high temporal resolution (LDHT) data is initially acquired during the early (arterial) phase of the contrast agent bolus arrival when rapid changes in signal intensity are expected. A second full-dose high spatial resolution (FDHS) dataset is then acquired during the later phases of the bolus arrival when the signal changes are slower and steadier. By combining these two sets of data, the LEGATOS method can improve the accuracy of quantitative analysis of tissue perfusion dynamics, permitting high spatial resolution assessment of tissue microvascular parameters. 

The LEGATOS method consists of two key steps. In the first step, a concatenated DTR 4D GBCA concentration volume is constructed containing a high temporal resolution ‘arterial’ phase followed by a later low temporal, high spatial resolution ‘parenchymal’ phase. The 4D LDHT DCE images are first co-registered to a baseline image frame of the FDHS DCE series to obtain a 4D LDHT_aligned_ volume. The signal intensity-time curves from this 4D LDHT_aligned_ and the 4D HS dynamic image volumes are then converted to GBCA concentration-time curves, before combining the HT and HS resolution series to construct a 4D GBCA concentration volume. In the second step, the LDHT_aligned_ arterial phase of each voxel concentration curve is re-scaled using a derived voxel-wise calibration ratio (ratio_calib_) to increase the spatial resolution of the derived kinetic parameter maps. Further details on the LEGATOS reconstruction method and derivation of the calibration ratio are provided in prior publications [20,21].

#### 1.1.2. Use of LEGATOS with the ETM and ET-CBF Model to Derive High Spatial Resolution K^trans^, v_p_, v_e_, and F_p_ Maps

The most widely used pharmacokinetic model in brain DCE-MRI is the ETM [18,19], which can be defined as follows:

(1) $$C_{t}(t)=v_{p}C_{p}(t)+K^{\mathrm{trans}}\;\int_{0}^{t}C_{p}(\tau )\exp(-\frac{K^{\mathrm{trans}}}{v_{e}}(t-\tau ))d\tau ,$$

where *C*_t_ is the tissue contrast agent concentration and *C*_p_ is the GBCA concentration in the vascular plasma space. In the convolution integral, *t* is considered a constant and *τ* is the variable. Through use of the LEGATOS technique in combination with the ETM high spatial resolution, estimates of K^trans^, v_p_, and v_e_ could be provided.

ET-CBF is a methodology for absolute CBF estimation, which uses data derived from a LDHT T1W DCE-MRI acquisition, and is based on the microsphere model and ET (early time points before the contrast agent has left the tissue) strategy [26]. The microsphere model can be written mathematically as follows:

(2) $$C_{t}(t)=f\cdot \int_{0}^{t}C_{b}(t\prime )dt\prime \;\mathrm{with}\;t\in \mathrm{ETW},\;$$

where the tissue GBCA concentration, *C*_t_(*t*), is equal to the amount of GBCA delivered to 1 mL of tissue by time *t*; *f* is the absolute CBF (mL min^−1^ mL^−1^); and *C*_b_(*t’*) is the arterial blood concentration at time *t*′. ETW is the early time window, i.e., the time window that meets the microsphere prerequisite. Using the microsphere model and Equation (2), low spatial resolution early time points absolute cerebral blood flow (CBF_ET_) maps can be derived from the arterial concentration-time curve of a low dose high temporal DCE-MRI acquisition.

High spatial resolution CBF_ET_ (CBF_ET-HS_) maps can also be derived from DTR DCE-MRI data through the use of the microsphere model with LEGATOS. CBF_ET-HS_ maps can be obtained either directly from the concatenated 4D high spatiotemporal resolution GBCA concentration volume generated using the LEGATOS approach described above or by using the calibration ratio maps generated as part of the LEGATOS procedure to rescale (see Equation (3)) the low spatial resolution CBF_ET_ (CBF_ET-HT_) derived from a 4D LDHT_aligned_ volume (see Appendix A for details):

(3) $${\mathrm{CBF}}_{\mathrm{ET}-\mathrm{HS}}={\mathrm{CBF}}_{\mathrm{ET}-\mathrm{HT}}\cdot {\mathrm{ratio}}_{\mathrm{calib}}.$$

#### 1.1.3. Theoretical Derivation of the Capillary Permeability-Surface Area Product (PS) from Derived K^trans^ and CBF_ET_ Values

Estimation of PS is based on the following relationship [38]:

(4) $$K^{\mathrm{trans}}=F_{p}\cdot \mathrm{PS}/(F_{p}+\mathrm{PS}),$$

where F_p_ is plasma flow. F_p_ relates to blood flow by the following:

(5) $$F_{p}={\mathrm{CBF}}_{\mathrm{ET}}\cdot (1-\mathrm{Hct}),\;$$

where Hct is the blood haematocrit and CBF_ET_ is the absolute cerebral blood flow estimated using the early time points method.

From Equation (4), we have the following:

(6) $$\mathrm{PS}=K^{\mathrm{trans}}/(1-K^{\mathrm{trans}}/F_{p}).$$

Therefore, PS can be calculated from the known K^trans^ and F_p_ using Equation (6). Using Equation (4), K^trans^ is dominated by the smallest of PS and F_p_ [38].

(7) $$K^{\mathrm{trans}}/\mathrm{PS}=F_{p}/(F_{p}+\mathrm{PS})=R_{\mathrm{Fp}}.$$

K^trans^/PS is the ratio of plasma flow to the sum of F_p_ and PS or R_Fp_, ranging from 0 to 1:

when F_p_ >> PS, R_Fp_ ≈ 1; 

when F_p_ = PS, R_Fp_ = 0.5; 

when F_p_ << PS, R_Fp_ becomes a small positive value (≈ F_p_/PS).

The R_Fp_ maps can be useful in identifying areas of high perfusion but inadequate permeability (High R_Fp_) and high permeability but inadequate perfusion (Low R_Fp_), respectively, thereby providing insight into tumour vascular heterogeneity [39]. High spatial resolution PS maps can also be obtained from estimates of K^trans^ and F_p_ derived using the LEGATOS_ETM_ and CBF_ET-HS_ techniques described above, respectively.

#### 1.1.4. Use of LEGATOS-Patlak for Measuring K^trans^ within Normal-Appearing Brain Regions

The Patlak model describes a highly perfused two-compartment tissue assuming unidirectional transport from the plasma into the extravascular extracellular space [6,40,41,42,43,44]. The GBCA concentration in tissue (*C*_t_) is given by the following:

(8) $$C_{t}(t)=v_{p}C_{p}(t)+K^{\mathrm{trans}}\int_{0}^{t}C_{p}(\tau )d\tau .$$

Dividing both sides of Equation (8) with *C*_p_(*t*), one obtains the Patlak plot (Equation (9)) where the slope represents K^trans^ and the intercept represents v_p_. The abscissa has the units of time, but this is not laboratory time; it is concentration-stretched time and will be referred to hereafter as t_stretch_ [41].

(9) $$\frac{C_{t}(t)}{C_{p}(t)}=v_{p}+K^{\mathrm{trans}}\frac{\int_{\;0}^{\;t}C_{p}(\tau )d\tau }{C_{p}(t)}.$$

One issue in the conventional Patlak plot method is that experimental errors are distorted when a non-linear model is transformed to a linear one [45]. This can introduce non-uniform (distorted) noise into the fitting procedure and reduce the reliability of the estimates of K^trans^ and v_p_. A previous study proposed a “hybrid FP-PP” method that combines a first-pass analytical approach [46,47] with the Patlak plot to address these limitations [24]. Both computer simulation and in vivo studies demonstrated improved reliability in v_p_ and K^trans^ estimates with the hybrid method [24], and further details on this hybrid FP-PP method can be found in the included reference [24].

The “hybrid FP-PP” model, however, requires high temporal resolution DCE-MRI data at the expense of limited spatial resolution and/or volume coverage. High spatial resolution assessment of low-level permeability in normal-appearing brain regions can be achieved by fitting the LEGATOS-generated 4D high spatiotemporal resolution GBCA concentration volumes with the hybrid FP-PP model. v_p_ estimates obtained from LEGATOS-ETM (instead of from the FP analysis) can also be used as the known v_p_ in the modified Patlak plot linear regression analysis [24] to produce the only free-fitting parameter, K^trans^.

**Figure 1:**
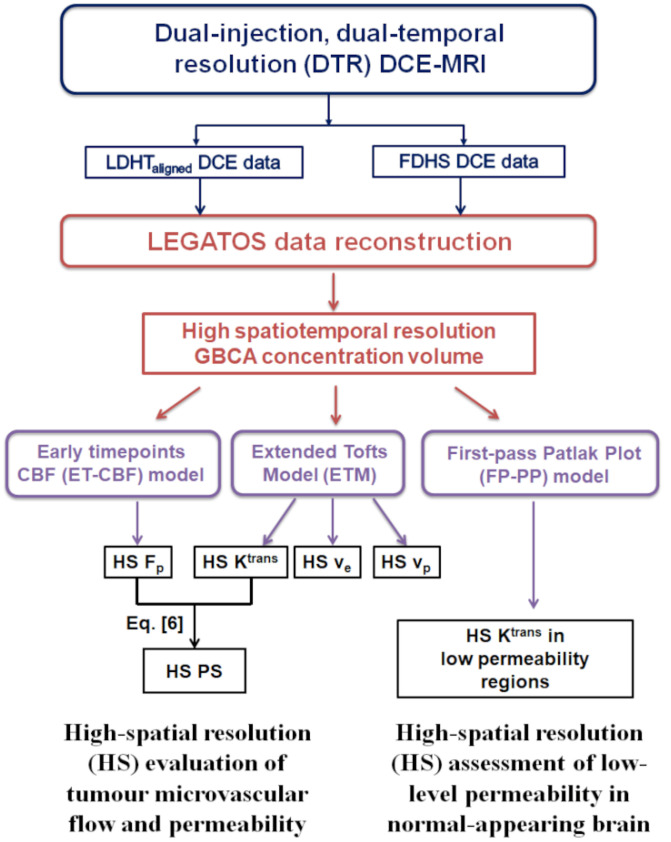
Flowchart showing the key steps in the multi-model LEGATOS integrated kinetic analysis. Blue highlights the key steps of the dual-injection, dual-temporal resolution DCE-MRI acquisition; red highlights the key steps of the LEGATOS data reconstruction to generate a high spatiotemporal resolution GBCA concentration volume; lilac highlights the key kinetic models used; and black highlights the derived microvascular parameters. ET-CBF = ‘early time points’ method for absolute cerebral blood flow quantification; HS = high spatial resolution; FDHS = full-dose high spatial resolution DCE-MRI data; K^trans^ = volume transfer constant between blood plasma and extravascular extracellular space; LDHT_aligned_ = low-dose high temporal resolution DCE-MRI data co-registered to the FDHS DCE series; F_p_ = plasma flow, where F_p_ = CBF_ET_·(1 − Hct); FP-PP = the hybrid first-pass Patlak plot model; GBCA= gadolinium-based contrast agent; LEGATOS = level and rescale the gadolinium contrast concentration curves of high temporal to high spatial DCE-MRI; PS = permeability-surface area product; v_e_ = volume of the extravascular extracellular space per unit volume of tissue; v_p_ = fractional blood plasma volume.

A flowchart in Figure 1 illustrates the key steps in the multi-model LEGATOS integrated pharmacokinetic analysis for the derivation of high spatial resolution microvascular biomarkers within both tumour and normal-appearing brain regions (see Appendix B for a list of abbreviations used in the dual-injection dual-temporal resolution DCE-MRI pharmacokinetic analysis). 

## 2. Results

### 2.1. High Spatial Multi-Model Assessment of Perfusion and Permeability Parameters within Both Tumour and Normal-Appearing Brain 

**Figure 2:**
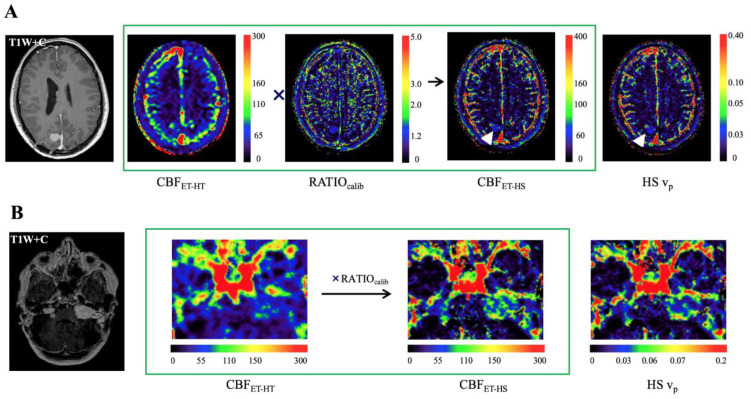
Use of the LEGATOS technique with the ‘early time points’ absolute cerebral blood flow (ET-CBF) method to generate high spatial resolution CBF_ET_ estimates. (**A**) Representative pre-treatment images from an NF2 patient with multiple meningiomas including a right parasagittal convexity meningioma (white arrow). (**B**) Representative images from a patient with bilateral NF2-related VS. Note the imaging artifact from the left-sided bone-anchored hearing aid within the post-contrast T1-weighted image. The low spatial resolution CBF_ET_ map (CBF_ET-HT_) derived from the low dose high temporal resolution DCE data is rescaled by the LEGATOS calibration ratio map to generate the high spatial resolution CBF_EHS_ map. T1W + C = T1-weighted image post-contrast.

Representative pre-treatment early time points absolute CBF_ET_ maps for a patient with NF2-related VS and meningioma are shown in Figure 2 and demonstrate how high spatial resolution absolute CBF_ET-HS_ maps can be achieved using the early time points model with LEGATOS. Figure 2A shows representative pre-treatment images from an NF2 patient with a small convexity meningioma (white arrow). This meningioma was not clearly defined within the native ET-CBF map (left) due to the low spatial resolution and partial volume effects, but it is much better demonstrated within the reconstructed HS-CBF map and the LEGATOS-ETM derived high spatial resolution v_p_ map (right column). 

Figure 2B shows representative images from a patient with bilateral NF2-related VS. The CBF_ET-HS_ map offered superior visualization of intratumoural heterogeneity within the left-sided VS compared to the CBF_ET-HT_ map and closely resembled the LEGATOS-ETM derived high spatial resolution tumour v_p_ map as expected. Compared to the unreconstructed, low spatial resolution CBF_ET_ images, the CBF_ET-HS_ maps offered superior visualization of both the smaller right-sided VS and intratumoural heterogeneity in blood flow. The CBF_ET-HS_ maps spatially corresponded with the pattern of vascularisation seen in the LEGATOS-ETM-derived high spatial resolution v_p_ maps.

**Figure 3:**
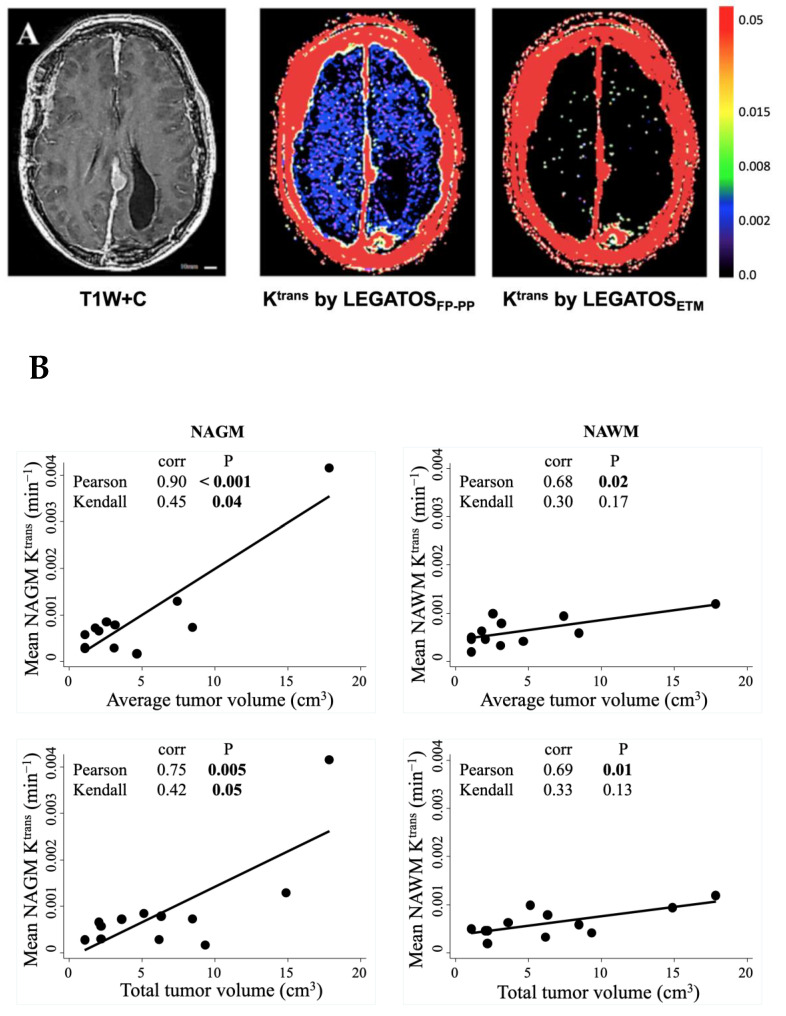
(**A**) Representative post-contrast T1W image and high spatial resolution K^trans^ maps in an NF2 patient with multiple meningioma. Data were fitted using either the hybrid FP-PP approach (LEGATOS_FP-PP_, middle) or ETM (LEGATOS_ETM_, right). Note the comparatively high K^trans^ values seen within the right-frontal and left-parasagittal meningioma on both maps. Compared to LEGATOS_ETM_ the LEGTOS_FP-PP_ permits high spatial resolution assessment of low-level vascular permeability within the normal-appearing brain. (**B**) Correlation between mean NAGM/NAWM K^trans^ and VS volume. Top row: correlation between average VS volume and mean NAGM (left)/NAWM (right) K^trans^ values; bottom row: correlation between cumulative VS volume and mean NAGM (left)/NAWM (right) K^trans^ values. NAGM = normal-appearing grey matter; NAWM = normal-appearing white matter. T1W + C = T1-weighted image post-contrast.

Representative high spatial resolution K^trans^ maps provided by the LEGATOS method, using either the FP-PP approach or ETM are shown in Figure 3A. Both maps showed comparably high K^trans^ values within the pre-treatment meningioma, with median tumour K^trans^ values of 0.025 and 0.017 min^−1^ for ETM and FP-PP models, respectively. The Patlak analysis underestimated K^trans^ of the meningioma because the backflux of GBCA from the extravascular extracellular space to the plasma was too large to ignore in the tumour tissue. Compared to the LEGATOS_ETM_ method, however, integration of the hybrid FP-PP model with LEGATOS (LEGATOS_FP-PP_) permitted high spatial resolution assessment of low-level K^trans^ in the normal-appearing brain.

Descriptive K^trans^ statistics obtained from NAGM/NAWM in the 12 patients are shown in Table 1. Segmented NAGM displayed non-significantly higher mean (*p* = 0.30) and significantly higher median (*p* = 0.03) and max (*p* = 0.002) K^trans^ values compared to segmented NAWM (paired t-test). Pearson correlation analysis (Figure 3B) shows that in this NF2 patient cohort, significant positive correlations between tumour volume and NAGM/NAWM K^trans^ values were observed. Mean K^trans^ measured from both the NAGM or NAWM segments showed a significant correlation with both average VS volume size (*p* ≤ 0.02) and total VS volume (*p* ≤ 0.01). Kendall rank correlation test also shows significant positive monotonic correlations of NAGM K^trans^ values with both average and total VS volume size (*p* = 0.04 and *p* = 0.05, respectively).

### 2.2. High Spatial Evaluation of Changes in Tumour Microvascular Parameters during Antiangiogenic Therapy

**Figure 4:**
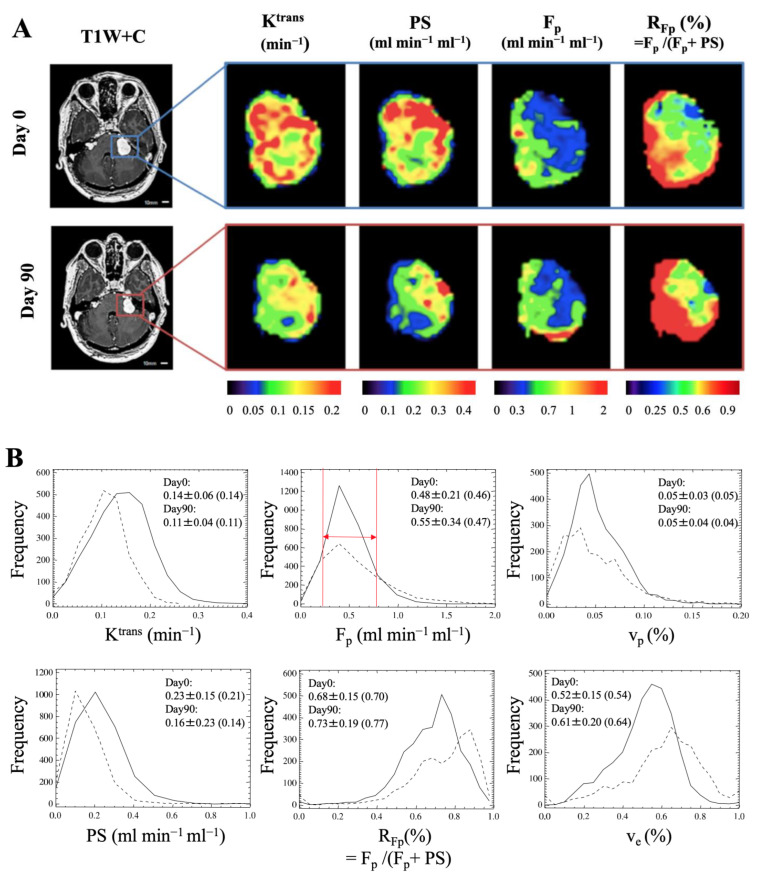
(**A**) High spatial resolution tumour K^trans^, PS, F_p_, and R_Fp_ (= F_p_/(F_p_ + PS)) maps imaged at day 0 (baseline) and day 90 in an NF2 patient with left-sided VS. (**B**) Histograms of tumour voxel values for K^trans^, F_p_, v_p_, PS, R_Fp_, and v_e_ at baseline (the solid line) and 90 days post-bevacizumab treatment (dashed line), calculated in the same tumour as in panel A. Tumour volume size was 6.01 cm^3^ (3005 voxels) at day 0 and 4.33 cm^3^ (2163 voxels) at day 90. The overall tumour volume was reduced by 28%, and tumour volume loss was principally seen in voxels with F_p_ in the range of 0.2 to 0.8 min^−1^ (red arrow lines) and v_p_ in the range of 0.02 to 0.10 in the histogram. Tumour mean ± SD (median) for each of the 6 parameters measured at day 0 and day 90 are shown in the panel corresponding to that parameter, respectively. T1W + C = T1-weighted image post-contrast. The x-axis of each histogram represents the range of parameter values divided into bins, and the y-axis represents frequency (how many voxels fall within each bin).

Representative pre- and post-bevacizumab high spatial resolution microvascular parameter maps (K^trans^, PS, F_p_, and R_Fp_) are shown in Figure 4A for an NF2 patient with a large VS. Pre-treatment, there was intratumoural heterogeneity in the derived perfusion and permeability metrics with distinct tumour regions showing either high F_p_ or high K^trans^ and PS, respectively. Such heterogeneity was also evident in derived R_Fp_ maps (ratio of plasma flow to the sum of F_p_ and PS, F_p_/(F_p_ + PS)), with regions showing high R_Fp_ (permeability limited) and low R_Fp_ (perfusion limited), respectively. At 90 days post-bevacizumab therapy, reductions in VS volume are also seen along with substantial reductions in both K^trans^ and PS. There is also an observed corresponding increase in R_Fp,_ with an increase in the tumour subregion displaying high perfusion but inadequate permeability.

In Figure 4B, the histogram distributions before and after 90 days of treatment, calculated for the same tumour, are shown. After 90 days of bevacizumab treatment, K^trans^ and PS histograms showed a shift towards lower values, with a median decrease of 21% in K^trans^ and a 33% decrease in PS. R_Fp_ histograms conversely showed a shift towards higher values, with a median increase of 10%. The overall tumour volume was reduced by 28%, and tumour volume loss was principally seen in voxels with F_p_ in the range of 0.2–0.8 min^−1^ (see *arrowed red lines* on F_p_ histogram), with 2638 out of 3005 and 1588 out of 2163 tumour voxels being in this range at day 0 and day 90 post-treatment, respectively. 

Post-treatment changes in each high spatial resolution microvascular parameter across the twelve imaged patients are shown in Table 2. Responding tumours displayed significant post-treatment reductions in K^trans^ (*p* ≤ 0.001) and PS (*p* = 0.0002) and significant increases in R_Fp_ (*p* = 0.004). Post-treatment decreases in F_p_ (*p* = 0.08), v_p_ (*p* = 0.07), and v_e_ (*p* = 0.56) were also seen but these did not reach statistical significance. Non-responding VS also displayed a significant increase in R_Fp_ at day 90 (*p* = 0.04). Decreases in mean K^trans^ and PS were not significant (*p* > 0.05); however, in contrast to responding tumours, non-responding VS displayed significant increases in v_e_ (*p* = 0.01). 

### 2.3. Differences between Responding and Non-Responding Tumours in Baseline (Pre-Treatment) High Spatial Resolution Microvascular Parameters

**Figure 5:**
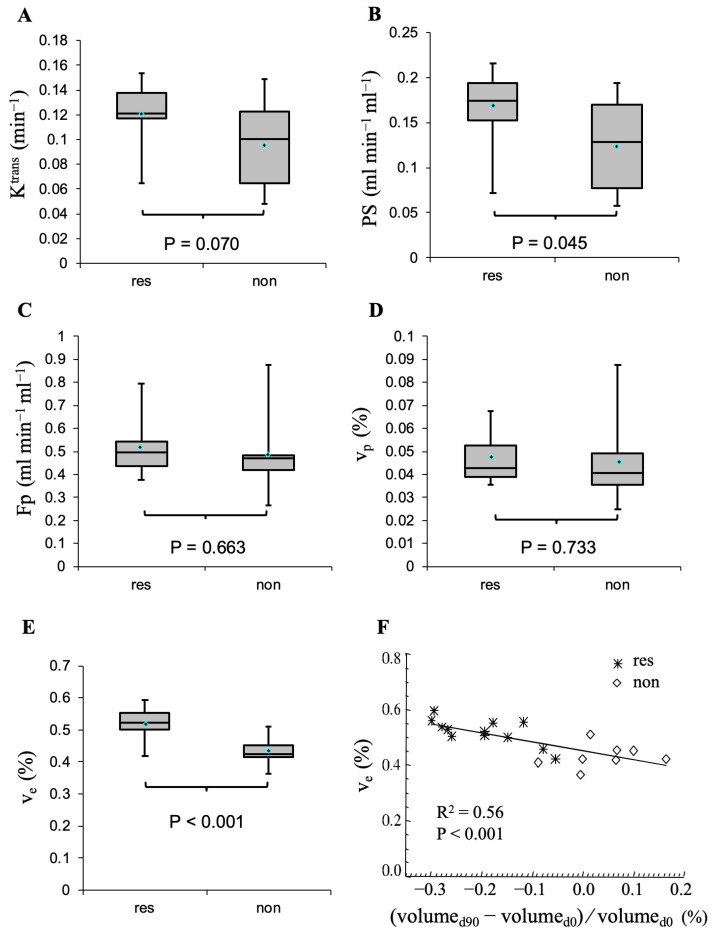
Predictive value of pre-treatment tumour median values of microvascular parameters. (**A**–**E**) Comparison of the pre-treatment (day 0) microvascular parameters (K^trans^, PS, Fp, v_p_, and v_e_) between the responder (res) and non-responder (non) VS groups. Boxplots show mean (dot on the box plot), median (bar within the box), upper and lower quartiles (box limits), and extreme values (whiskers). The *p* values are calculated using a two-sided Student *t*-test. (**F**) Correlation between pre-treatment tumour median v_e_ and percentage change in tumour volume at day 90; volume_d0_ = baseline tumour volume; volume_d90_ = tumour volume at day 90.

Differences in pre-treatment high spatial microvascular parameters between responding and non-responding VS are shown in Figure 5. Responding tumours showed significantly higher pre-treatment PS (*p* = 0.045) and v_e_ (*p* < 0.001) values. Responding tumours also displayed higher K^trans^ than non-responding VS but these differences (*p* = 0.07) did not reach statistical significance. Of the five parameters, baseline v_e_ correlated most strongly with percentage volume change at day 90 (*p* < 0.001). 

Table 3 shows associations between the estimated pre-treatment tumour median K^trans^, Fp, PS, v_p_, and v_e_ and tumour volumetric parameters (the baseline tumour volume, tumour volume change, or percentage tumour volume change at day 90) assessed by linear regression analysis. Both PS and K^trans^ (a hybrid flow-permeability parameter) showed a significant correlation with baseline tumour volume (*p* = 0.03 and *p* = 0.05, respectively) and a non-significant trend of correlation with tumour volume change at day 90 (*p* = 0.06 and *p* = 0.12, respectively). On the other hand, v_e_, an oedema-associated parameter [48], showed a significant correlation with tumour volume change and percentage tumour volume change at day 90 (*p* = 0.04 and *p* = 0.0002, respectively) and a non-significant trend of correlation with baseline tumour volume (*p* = 0.08). F_p_ and v_p_ did not show correlations with any of the three tumour volumetric parameters.

Univariate logistic regression analysis revealed that, of the five microvascular parameters derived using the new method, v_e_ estimates (AUC = 0.896, *p* = 0.02, Table 4) showed the greatest ability in the prediction of 90-day response in NF2-related vestibular schwannoma. v_p_ was the only variable with a *p* value greater than 0.50. In multivariate analysis, the backward selection procedure starts with the five potential predictors and eliminates the least significant variable at each step. The model with three variables (v_e_, PS, and F_p_) demonstrated a higher AUC, sensitivity, and specificity than the models with fewer variables.

## 3. Discussion

We have described a novel processing technique for DTR DCE-MRI, which, using the LEGATOS method with multi-model kinetic analysis, allows for the derivation of accurate, high spatial resolution, whole-brain coverage kinetic parameter maps. In contrast to the use of the extended Tofts model alone, this new approach allows for the separate derivation of tumoural blood flow and permeability, as well as a high spatial resolution assessment of low-level permeability in a normal-appearing brain. We evaluated our new analysis method through an in vivo study of patients with neurofibromatosis type 2 (NF2)-related vestibular schwannoma undergoing antiangiogenic (bevacizumab) therapy.

In a previous longitudinal DCE-MRI study of NF2-related VS, it was demonstrated that pre-treatment, low spatial resolution tumoural K^trans^ estimates had the potential ability to predict later VS volume response to anti-VEGF therapy [4]. As a parameter, however, K^trans^ reflects regional capillary blood flow, capillary endothelial permeability, and the surface area of the capillary endothelial membrane [22], and the extent to which changes in each of these physiological variables changed during treatment was unknown. From earlier in vivo studies, the primary action of VEGF inhibitors, such as bevacizumab, was thought to be the rapid reduction in capillary endothelial membrane permeability [49,50], and it was hypothesized that the reduction in K^trans^ observed following treatment might be indicative of this mechanism. However, an alternative interpretation was that changes in tumour blood flow could also affect measured K^trans^, and to address this question, our study utilized a high spatial resolution multi-kinetic model analysis technique that permits simultaneous estimation of both F_p_ and PS within the tumour microvasculature. 

Our results demonstrated that the capillary permeability-surface area product (PS) showed more pronounced treatment-related changes than K^trans^, with responding tumours showing both significantly higher baseline PS and significant reductions in PS at 90 days post-treatment compared to non-responding VS. On the other hand, our findings revealed that F_p_ alone was not predictive of tumour volume response to bevacizumab treatment. There was no difference in pre-treatment F_p_ between responding and non-responding tumour groups, and in neither group was there a significant change in F_p_ during treatment observed. Instead, across all VS studied, there was an increase in the relative scale of perfusion to permeability (higher R_Fp_) during treatment. Overall, these imaging observations suggest that the changes observed in K^trans^ following bevacizumab treatment are primarily driven by a reduction in capillary endothelial membrane permeability, rather than hypothesized changes in tumour blood flow. Such a finding is in line with previous in vivo murine models evaluating anti-VEGF therapy response [4,51,52], which have shown early (< 24 h) vascular normalization with reductions in both vascular permeability and the surface area of vessels; this is to our knowledge the first time that microvascular flow and permeability changes following anti-VEGF therapy in NF2-related VS have been differentiated through an in vivo human imaging analysis. 

This study demonstrated that, of the five microvascular parameters investigated (K^trans^, PS, F_p_, v_p_, and v_e_), baseline v_e_ showed the most significant association with tumour volume response to bevacizumab treatment. These findings are supported by previous studies on the predictive value of an apparent diffusional coefficient (ADC) and a native longitudinal relaxation rate (R1_N_) in relation to tumour volume response to the VEGF inhibition [4,35,53]. High values of ADC and low values of R1_N_ are in keeping with a larger extravascular extracellular space, increased levels of interstitial free fluid, and likely higher capillary permeability. Within our study, we found that baseline v_e_ had a strong correlation with percent volume reduction at day 90, which is consistent with Plotkin et al., who found that high baseline ADC values were associated with tumour volume reduction at 3 months [35]. Although high baseline v_e_ was a strong correlate of tumour shrinkage, tumour median values of v_e_ showed only a non-significant percentage decrease (−3.1% ± 21.4%, *p* = 0.56) in the ‘responder’ group and a significant percentage increase (19.3% ± 17.0%, *p* = 0.01) in the ‘non-responder’ group at day 90. A multivariate model combining pre-treatment v_e_, PS, and F_p_ demonstrated high sensitivity and specificity for the prediction of volumetric response at 90 days, and these results, whilst preliminary, suggest that initial volumetric response to anti-VEGF therapy appears more likely in VSs that show both high levels of permeability and intratumoural oedema [4,54]. Future larger studies are, however, required to better understand the role of v_e_ and PS in anti-VEGF therapy prediction. 

Alongside demonstrating that the presented multi-model analysis approach permits differentiation of flow and permeability effects within the tumour microenvironment, we demonstrated that integration of the hybrid FP-PP model with LEGATOS also permits high spatial resolution assessment of low-level permeability within the normal-appearing brain. In keeping with previous literature demonstrating higher blood volume, higher vascular surface area, and higher transfer constant estimates in grey compared to white matter [15,55,56], our study data demonstrated higher K^trans^ estimates within NAGM compared to NAWM. In this NF2 patient cohort, a significant positive correlation between total VS disease burden and K^trans^ values in NAGM/NAWM was observed. In patients with VS, there is emerging evidence that there can be effects on the brain remote to the tumour, with previous diffusion and functional MRI studies demonstrating widespread changes in activity networks, grey matter volume, and white matter fibre integrity in auditory and non-auditory regions in patients with these tumours [57,58,59,60,61]. To date, however, changes in DCE-MRI parameters within the normal-appearing brain of patients with NF2 have not been evaluated and further large, detailed studies are required to better understand the pathophysiology of the observed K^trans^ changes and their relationship to the tumour burden in these patients. 

A key limitation of the presented study is that the number of patient participants was small. The primary aim of this study was to develop and evaluate the in vivo applicability of a high spatial resolution multi-kinetic model DCE-MRI approach rather than robustly evaluate predictors of NF2-related VS antiangiogenic response through a large clinical trial. Patients with NF2-related VS undergoing bevacizumab therapy were used as a disease model to test this new analysis approach due to both the previously reported predictive potential of DCE-MRI in this tumour group and the purported vascular normalization mechanism of anti-VEGF therapy that has been demonstrated in pre-clinical studies, which could be interrogated through our DCE-MRI approach. Larger future prospective studies incorporating this multi-model DCE-MRI analysis should be performed to better understand the predictive potential of each derived parameter. A second limitation is that the DTR DCE-MRI protocol and analysis technique used in the study has relatively long scan (> 10 min) and data processing times. Using the multi-model LEGATOS integrated pharmacokinetic analysis technique described here, the total computer processing time (excluding image registration time) to obtain the comprehensive panel of microvascular biomarkers with high spatial resolution and whole-brain coverage was approximately six hours. The potential impact of MRI data noise on the reliability of derived microvascular parameters also potentially remains a challenge. Further research and optimization are required to overcome these limitations and establish the technique’s practical value in routine clinical practice. 

## 4. Materials and Methods

### 4.1. Patients

As a disease model to evaluate this new multi-model DCE-MRI analysis technique, previously acquired dual-injection, DTR DCE-MRI data in twelve consecutive patients with NF2-related VS were analysed. All patients were undergoing antiangiogenic therapy with the anti-VEGF antibody, bevacizumab (Avastin©), and across all patients there were twenty VSs, with four patients having undergone previous surgical resection of a VS. All patients had been recruited and scanned as part of an earlier described study investigating MR imaging predictors of antiangiogenic response [4]. Patients with standard contraindications to MR imaging or contrast agent administration were excluded. Only patients with proven NF2 and at least one VS demonstrating a high growth rate (defined as an annual increase of 4 mm or greater in the maximal transverse diameter) were included. Detailed study inclusion/exclusion criteria along with bevacizumab dose information are detailed in prior publications [4]. Ethical approval was in place for this study (REC reference 13/NW/0247) and all recruited participants had previously consented to later analysis of their MRI data.

Patients underwent DCE-MRI on 2 occasions: pre-treatment (day 0) and 3 months (day 90) following treatment. Tumours (VSs) were classified according to volumetric response/reduction over the 90-day bevacizumab treatment period with response defined as a volume reduction exceeding 0.125 cm^3^ and/or a relative volume decrease exceeding 5%. Further details on how volume response was defined are provided in the included reference [4]. 

### 4.2. MRI Data Acquisition

All patients were imaged at 1.5 T (Philips Achieva, Best, The Netherlands) and dual-injection DTR DCE-MRI data were collected using a 3D spoiled gradient-recalled echo sequence as described previously [62]. Using a power injector, a macrocyclic GBCA (gadoterate meglumine; Dotarem, Guerbet S.A., Villepinte, France) was administered at a rate of 3 mL/s as an intravenous bolus, followed by a chaser of 20 mL/s of 0.9% saline at the same rate. A high temporal (Δ*t* = 1.0 s) but low spatial (voxel size = 2.5 × 2.5 × 6.35 mm^3^) resolution DCE-MRI series (N_Frame_ = 300) with a low-dose fixed-volume (3 mL) of GBCA was performed for a total scan duration of 5.1 min. Subsequently, a high spatial (voxel size = 1 × 1 × 2 mm^3^) but low temporal (Δ*t* = 10.7 s) resolution DCE-MRI series with a full dose (0.2 mL/kg·weight—3 mL dose of pre-bolus) of GBCA was performed for a total scan duration of 10.6 min (N_Frame_ = 60). Variable flip angle (a = 2°, 8°, 15°, and 20°) acquisitions were performed prior to the LDHT and FDHS DCE series for baseline longitudinal relaxation rate estimation.

### 4.3. Image Processing

A 4D high spatiotemporal resolution GBCA concentration volume was generated from DTR DCE-MRI using the LEGATOS technique. The LEGATOS reconstructed concentration volume was subsequently fitted with the ETM, ET-CBF, and hybrid FP-PP models to derive high spatial resolution estimates of K^trans^, v_p_, v_e_, CBF_ET_, F_p_, R_Fp_, and PS, and to detect low-level permeability (K^trans^) in normal-appearing brain regions. 

As outlined above, low spatial resolution CBF_ET_ maps were derived from the arterial phase of the 4D LDHT_aligned_ volume (which is the arterial phase of the concatenated 4D DTR GBCA concentration volume generated in key step I of the LEGATOS approach). High spatial resolution CBF_ET_ maps were obtained by rescaling the low spatial resolution CBF_ET_ with the calibration ratio maps generated as part of the LEGATOS procedure. CBF_ET-HS_ and CBF_ET-HT_ maps could therefore be compared as part of this study. 

Quantifying DCE-MRI-derived kinetic parameters through modelling requires a suitable vascular input function (VIF), and previous studies have demonstrated that the usage of the superior sagittal sinus (SSS) as a surrogate input function provides a good approximation [63,64]. This VIF measurement method uses a semi-automatic extraction method to identify voxels within the SSS that display maximum enhancement during the first pass of the GBCA bolus, as has been previously described [65]. For the LEGATOS reconstruction, a combined vascular input function is constructed through concatenation of the GBCA concentration-time curve, *C*_p_ (*t*), from the LDHT series with the later parenchymal phase of the dose-calibrated GBCA concentration-time curve from the FDHS-DCE series [4,6]. The amplitude of the *C*_p_ (*t*) from the FDHS series is scaled down to match the LDHT-derived *C*_p_ (*t*) using the dose calibration ratio prior to concatenation. Further details on the derivation of the VIF for this analysis can be found in the included reference [20].

SPM (statistical parametric mapping) [66] was used for all image co-registration and for segmentation of the 3D T1W MRI data into normal-appearing grey matter (NAGM), white matter (NAWM), and cerebrospinal fluid (CSF) probability maps. After re-alignment/re-slicing of each map to a baseline FDHS DCE frame, masks of NAGM and NAWM were generated using a probability cut-off of 0.95. Only supratentorial NAGM/NAWM regions were included in each mask for quantitative analysis of K^trans^ within normal-appearing brain regions. 

### 4.4. Statistical Analysis

High spatial resolution tumour microvascular parameters derived using the above multi-model (ETM, ET-CBF, FP-PP) approach were compared both before and 90 days after antiangiogenic therapy. Median tumour values for K^trans^, PS, F_p_, R_Fp_, v_e_, and v_p_ were calculated for each visit for the 20 VSs across the 12 NF2 patients. The group mean and standard deviation of these tumour median values were compared across the two visits using a paired t-test, with responding (N = 12) and non-responding (N = 8) VSs analysed separately. Histograms of the fitted voxelwise microvascular parameters (K^trans^, PS, F_p_, R_Fp_, v_e_, and v_p_) before and 90 days after treatment were also compared.

Estimates of K^trans^ within NAGM and NAWM across the twelve patients were compared using descriptive statistics and paired *t*-tests. Estimates of mean K^trans^ measured from the NAGM/NAWM segmentations were also compared with VS volume using scatterplot analysis and reported as Pearson’s and Kendall’s correlation coefficients (r, r_s_, and τ, respectively). The tumour volume used in the correlation analysis was defined as either (1) the average size across both VSs (or the size of the single VS in patients previously having undergone a VS resection) or (2) the cumulative tumour volume across both/single VSs. 

The hypothesis that pre-treatment tumour median values of K^trans^, PS, F_p_, v_p_, and v_e_ do not differ between responding and non-responding VS was tested using the unpaired Student’s *t*-test. Binary logistic regression (S-Plus, version 6.1; Insightful, Seattle, WA, USA) was also performed to assess the ability of these imaging biomarkers to predict tumour response at day 90. The predictive performance of the model was assessed by receiver operator characteristics (ROC) analysis and calculation of the area under the curve (AUC). Univariate and multivariate analyses were performed. A backward elimination variable selection method was used in the multivariate analysis to obtain the best model.

Linear regression analysis was also performed to assess the relationship between imaging parameters at baseline and tumour volumetric parameters (the baseline tumour volume, tumour volume change, or percentage tumour volume change at day 90). A *p* value less than 0.05 was considered to indicate a statistically significant difference.

## 5. Conclusions

This study proposed a new DTR DCE-MRI processing technique for high spatial resolution interrogation of flow and permeability effects within both the tumour microenvironment and the associated normal-appearing brain regions. Evaluation in a cohort of patients with NF2-related VS undergoing anti-VEGF therapy demonstrated that there was an association between baseline tumour volume and low-level permeability (K^trans^) changes within the normal-appearing brain and that, within the tumour microenvironment, this new approach allowed for concomitant evaluation of blood flow and permeability changes, with the tumoural capillary permeability-surface area product demonstrating the most pronounced reduction at 90 days. In a preliminary study of volumetric response predictors, baseline v_e_ showed the strongest correlation with the change in tumour volume during treatment, and a multivariate model combining v_e_, PS, and F_p_ demonstrated high sensitivity and specificity for the prediction of volume reduction at 90 days. These results highlight the utility of this novel DCE-MRI analysis approach in evaluating tumour microvascular changes during treatment and the need for future larger studies investigating its role in predicting antiangiogenic therapy response.

## Figures and Tables

**Table 1:** K^trans^ statistics estimated from segmented normal-appearing grey matter (NAGM) and white matter (NAWM) in twelve patients with NF2.

K^trans^ Statistics of NAGM/NAWM Segments	NAGM Segment Statistics Mean ± SD	NAWM Segment Statistics Mean ± SD	*p* Value Paired *t*-Test
Mean (min^−1^)	0.00089 ± 0.0011	0.00062 ± 0.00030	0.30
Median (min^−1^)	0.00047 ± 0.00037	0.00028 ± 0.00034	**0.03**
SD (min^−1^)	0.0030 ± 0.0026	0.0012 ± 0.00021	**0.04**
Max (min^−1^)	0.050 ± 0.034	0.014 ± 0.0058	**0.002**
Min (min^−1^)	−0.023 ± 0.0074	−0.015 ± 0.0040	**0.002**

Significant *p* values are shown in bold type.

**Table 2:** Mean and standard deviation of the tumour median K^trans^, F_p_, PS, R_Fp_, v_p_, and v_e_ estimated at day 0 and day 90 from 20 VSs in 12 NF2 patients.

Microvascular Parameter	Day 0 Mean ± SD (Median)	Day 90 Mean ± SD (Median)	*p* Value Paired *t*-Test
**K^trans^ (min^−1^)**			
Res (N = 12)	0.121 ± 0.023 (0.121)	0.083 ± 0.031 (0.082)	**0.001**
Non (N = 8)	0.095 ± 0.037 (0.100)	0.086 ± 0.025 (0.096)	0.18
All (N = 20)	0.111 ± 0.059 (0.123)	0.085 ± 0.028 (0.093)	**0.0008**
**PS (mL min^−1^ mL^−1^)**			
Res (N = 12)	0.169 ± 0.039 (0.174)	0.109 ± 0.043 (0.106)	**0.0002**
Non (N = 8)	0.125 ± 0.053 (0.128)	0.107 ± 0.032 (0.118)	0.10
All (N = 20)	0.151 ± 0.087 (0.162)	0.108 ± 0.038 (0.117)	**0.0001**
**F_p_ (mL min^−1^ mL^−1^)**			
Res (N = 12)	0.514 ± 0.114 (0.496)	0.414 ± 0.103 (0.423)	0.08
Non (N = 8)	0.485 ± 0.175 (0.470)	0.541 ± 0.139 (0.579)	0.36
All (N = 20)	0.502 ± 0.270 (0.477)	0.465 ± 0.132 (0.452)	0.37
**R_Fp_ (%)**			
Res (N = 12)	0.752 ± 0.065 (0.744)	0.792 ± 0.048 (0.788)	**0.004**
Non (N = 8)	0.784 ± 0.063 (0.790)	0.826 ± 0.045 (0.820)	**0.04**
All (N = 20)	0.765 ± 0.114 (0.761)	0.806 ±0.049 (0.799)	**0.0003**
**v_p_ (%)**			
Res (N = 12)	0.047 ± 0.012 (0.043)	0.038 ± 0.009 (0.040)	0.07
Non (N = 8)	0.045 ± 0.019 (0.040)	0.045 ± 0.010 (0.047)	0.94
All (N = 20)	0.046 ± 0.025 (0.042)	0.040 ± 0.010 (0.041)	0.13
**v_e_ (%)**			
Res (N = 12)	0.519 ± 0.047 (0.523)	0.498 ± 0.103 (0.457)	0.56
Non (N = 8)	0.431 ± 0.042 (0.423)	0.511 ± 0.063 (0.500)	**0.01**
All (N = 20)	0.484 ± 0.259 (0.500)	0.504 ± 0.196 (0.488)	0.43

Abbreviations: Res = responders, Non = non-responders. Significant *p* values are shown in bold type.

**Table 3:** Correlations between tumour median K^trans^, Fp, PS, v_p_, and v_e_ estimated at day 0 and tumour volumetric parameters.

Linear Regression Analysis (N = 20)	Tumour Volume (cm^3^; Day 0)	Tumour Volume Change (cm^3^; Day 90)	Percentage Tumour Volume Change (%; Day 90)
K^trans^ (min^−1^)	**R^2^ = 0.19** ***p* = 0.05**	R^2^ = 0.13 *p* = 0.12	R^2^ = 0.08 *p* = 0.23
PS (mL min^−1^ mL^−1^)	**R^2^ = 0.24** ***p* = 0.03**	R^2^ = 0.18 *p* = 0.06	R^2^ = 0.10 *p* = 0.18
Fp (mL min^−1^ mL^−1^)	R^2^ = 0.01 *p* = 0.71	R^2^ = 0.01 *p* = 0.67	R^2^ = 0.01 *p* = 0.66
v_p_ (%)	R^2^ = 0.02 *p* = 0.55	R^2^ = 0.03 *p* = 0.48	R^2^ = 0.00 *p* = 0.90
v_e_ (%)	R^2^ = 0.16 *p* = 0.08	**R^2^ = 0.21** ***p* = 0.04**	**R^2^ = 0.56** ***p* < 0.001**

Bold type indicates statistically significant *p* ≤ 0.05.

**Table 4:** Area under the receiver operator curve, sensitivity, and specificity of the binomial regression model for the prediction of tumour response using univariate (**A**) and multivariate analysis (**B**).

Prediction of Response	AUC-ROC (*p* Value)	Sensitivity	Specificity	Overall Classification
**A. Univariate analysis**				
v_e_ (%)	0.896 (0.024)	0.830	0.875	0.850
PS (mL min^−1^ mL^−1^)	0.708 (0.10)	0.920	0.500	0.750
K^trans^ (min^−1^)	0.688 (0.11)	0.92	0.375	0.700
F_p_ (mL min^−1^ mL^−1^)	0.667 (0.50)	1.00	0.125	0.650
v_p_ (%)	0.615 (0.61)	-	-	-
**B. Multivariate analysis with backward selection**				
Step 1 v_e_ + PS + K^trans^ + F_p_ + v_p_	0.948 (0.031; 0.92; 0.93; 0.69; 0.83)	0.830	0.875	0.850
Step 2 v_e_ + PS + F_p_ + v_p_	0.948 (0.030; 0.31; 0.70; 0.78)	0.830	0.875	0.850
Step 3 v_e_ + PS + F_p_	0.948 (0.032; 0.35; 0.70)	0.830	0.875	0.850
Step 4 v_e_ + PS	0.938 (0.036; 0.37)	0.830	0.750	0.800
Step 5 v_e_	0.896 (0.024)	0.830	0.875	0.850

## Data Availability

The datasets generated during and/or analysed during the current study are available from the corresponding author upon reasonable request.

## Appendix A

Measurement of CBF_ET_ has been previously described and is based on averaging the concentration of early time points during the first pass of a low-dose compact bolus [26]. For dual-injection, dual-temporal resolution DCE-MRI, high spatial resolution CBF_ET_ (CBF_ET-HS_) maps can also be derived through rescaling the low spatial resolution CBF_ET_ (CBF_ET-HT_) derived from the 4D LDHT_aligned_ volume with the calibration ratio maps generated as part of the LEGATOS procedure. Assuming that the GBCA concentration curve in each HS pixel has a similar first pass shape as its concatenated LDHT_aligned_ arterial phase, the ratio of the HS arterial phase, *C*_t-HS_(*t*), over the LDHT_aligned_ arterial phase, *C*_t-HT_(*t*), can be used to convert the CBF estimate derived from the LDHT_aligned_ arterial phase (CBF_ET-HT_) to the CBF of the concatenated HS pixel, CBF_ET-HS_, that is,

(A1) $${\mathrm{CBF}}_{\mathrm{ET}-\mathrm{HS}}/{\mathrm{CBF}}_{\mathrm{ET}-\mathrm{HT}}=C_{t-\mathrm{HS}}(t)/C_{t-\mathrm{HT}}(t),\;\;t\in \mathrm{arterial}\;\mathrm{phase}.$$

Based on the above, the calibration ratio, ratio_calib_, can be defined as follows:

(A2) $${\mathrm{ratio}}_{\mathrm{calib}}=C_{t-\mathrm{HS}}(t_{\mathrm{adj}})/C_{t-\mathrm{HT}}(t_{\mathrm{adj}})={\mathrm{CBF}}_{\mathrm{ET}-\mathrm{HS}}/{\mathrm{CBF}}_{\mathrm{ET}-\mathrm{HT}},$$

where *t*_adj_ is the ending time point of the arterial phase. In the proposed LEGATOS method, *C*_t-HS_(t_adj_) was calculated as the mean concentration of the five HS frames following the HT arterial phase and *C*_t-HT_(*t*_adj_) as the mean concentration of several ending frames of the HT arterial phase series.

The derived 3D calibration ratio map from Equation (A2) can then be used to convert a low spatial resolution CBF map derived from the LDHT_aligned_ arterial phase to high spatial resolution:

(A3) $${\mathrm{CBF}}_{\mathrm{ET}-\mathrm{HS}}={\mathrm{CBF}}_{\mathrm{ET}-\mathrm{HT}}\cdot {\mathrm{ratio}}_{\mathrm{calib}}.$$

## Appendix B

List of abbreviations used in dual-injection dual-temporal resolution DCE-MRI pharmacokinetic analysis.

**Symbol**	**Definition**	**Units**
CBF	Cerebral blood flow	mL min^−1^ mL^−1^
CBF_ET_	Absolute cerebral blood flow generated using early time points method	mL min^−1^ mL^−1^
CBF_ET-HS_	High spatial resolution estimates of CBF_ET_	mL min^−1^ mL^−1^
CBF_ET-HT_	Low spatial resolution estimates of CBF_ET_	mL min^−1^ mL^−1^
*C*_b_(*t*)	Concentration of contrast medium in arterial blood at time t	mmol
*C*_p_	Concentration of contrast medium in arterial blood plasma at time t	mmol
*C*_t_(*t*)	Concentration of contrast medium in the voxel at time t	mmol
DCE-MRI	Dynamic contrast-enhanced MRI	
DTR	Dual-temporal resolution	
ETM	The extended Tofts model	
ET-CBF	The ‘early time points’ method for absolute cerebral blood flow quantification	
FP-PP	The hybrid first-pass Patlak plot method	
ETW	The early time window, i.e., the time window that meets the microsphere prerequisite.	
FDHS	Full-dose high spatial resolution	
F_p_	Plasma flow	mL min^−1^ mL^−1^
HS	High spatial (HS) resolution	
HT	High temporal (HT) resolution	
K^trans^	Volume transfer constant between blood plasma and extravascular extracellular space	min^−1^
LDHT	Low-dose high temporal resolution	
LDHT_aligned_	4D LDHT DCE images co-registered to subsequent HS DCE series in dual-injection DTR DCE-MRI	
LEGATOS	A DTR DCE-MRI data construction technique: the level and rescale the gadolinium contrast concentration curves of high temporal to high spatial	
R_Fp_	The ratio of F_p_ to the sum of F_p_ and PS	None
PS	Capillary permeability–surface area product per unit mass of tissue	mL min^−1^ mL^−1^
v_e_	Volume of the extravascular extracellular space per unit volume of tissue	none
v_p_	Fractional blood plasma volume	none

## References

[1] O’Connor J.P., Jackson A., Parker G.J., Roberts C., Jayson G.C. (2012). Dynamic contrast-enhanced MRI in clinical trials of antivascular therapies. Nat. Rev. Clin. Oncol..

[2] Essig M., Anzalone N., Combs S.E., Dorfler A., Lee S.K., Picozzi P., Rovira A., Weller M., Law M. (2012). MR imaging of neoplastic central nervous system lesions: Review and recommendations for current practice. AJNR Am. J. Neuroradiol..

[3] Jain R. (2013). Measurements of tumor vascular leakiness using DCE in brain tumors: Clinical applications. NMR Biomed..

[4] Li K.-L., Djoukhadar I., Zhu X., Zhao S., Lloyd S., McCabe M., McBain C., Evans D.G., Jackson A. (2016). Vascular biomarkers derived from dynamic contrast-enhanced MRI predict response of vestibular schwannoma to antiangiogenic therapy in type 2 neurofibromatosis. Neuro-oncology.

[5] Lewis D., Roncaroli F., Agushi E., Mosses D., Williams R., Li K.-L., Zhu X., Hinz R., Atkinson R., Wadeson A. (2019). Inflammation and vascular permeability correlate with growth in sporadic vestibular schwannoma. Neuro-oncology.

[6] Larsson H.B., Courivaud F., Rostrup E., Hansen A.E. (2009). Measurement of brain perfusion, blood volume, and blood-brain barrier permeability, using dynamic contrast-enhanced T(1)-weighted MRI at 3 tesla. Magn. Reson. Med..

[7] Van Dijken B.R.J., van Laar P.J., Smits M., Dankbaar J.W., Enting R.H., van der Hoorn A. (2019). Perfusion MRI in treatment evaluation of glioblastomas: Clinical relevance of current and future techniques. J. Magn. Reson. Imaging.

[8] Lewis D., Donofrio C.A., O’leary C., Li K.-L., Zhu X., Williams R., Djoukhadar I., Agushi E., Hannan C.J., Stapleton E. (2021). The microenvironment in sporadic and neurofibromatosis type II–related vestibular schwannoma: The same tumor or different? A comparative imaging and neuropathology study. J. Neurosurg..

[9] Jackson A., O’Connor J.P., Parker G.J., Jayson G.C. (2007). Imaging Tumor Vascular Heterogeneity and Angiogenesis using Dynamic Contrast-Enhanced Magnetic Resonance Imaging. Clin. Cancer Res..

[10] O’Connor J.P.B., Jackson A., Parker G.J.M., Jayson G.C. (2007). DCE-MRI biomarkers in the clinical evaluation of antiangiogenic and vascular disrupting agents. Br. J. Cancer.

[11] Ingrisch M., Sourbron S., Morhard D., Ertl-Wagner B., Kumpfel T., Hohlfeld R., Reiser M., Glaser C. (2012). Quantification of perfusion and permeability in multiple sclerosis: Dynamic contrast-enhanced MRI in 3D at 3T. Investig. Radiol..

[12] Rose C.J., O’Connor J.P., Cootes T.F., Taylor C.J., Jayson G.C., Parker G.J., Waterton J.C. (2014). Indexed distribution analysis for improved significance testing of spatially heterogeneous parameter maps: Application to dynamic contrast-enhanced MRI biomarkers. Magn. Reson. Med..

[13] Mallio C.A., Rovira À., Parizel P.M., Quattrocchi C.C. (2020). Exposure to gadolinium and neurotoxicity: Current status of preclinical and clinical studies. Neuroradiology.

[14] Gulani V., Calamante F., Shellock F.G., Kanal E., Reeder S.B. (2017). Gadolinium deposition in the brain: Summary of evidence and recommendations. Lancet Neurol..

[15] Cramer S., Simonsen H., Frederiksen J., Rostrup E., Larsson H. (2014). Abnormal blood–brain barrier permeability in normal appearing white matter in multiple sclerosis investigated by MRI. Neuroimage Clin..

[16] Kershaw L.E., Buckley D.L. (2006). Precision in measurements of perfusion and microvascular permeability withT1-weighted dynamic contrast-enhanced MRI. Magn. Reson. Med..

[17] Barnes S.R., Ng T.S.C., Montagne A., Law M., Zlokovic B.V., Jacobs R. (2015). Optimal acquisition and modeling parameters for accurate assessment of low Ktrans blood-brain barrier permeability using dynamic contrast-enhanced MRI. Magn. Reson. Med..

[18] Tofts P.S. (1997). Modeling tracer kinetics in dynamic Gd-DTPA MR imaging. J. Magn. Reson. Imaging.

[19] Fritz-Hansen T., Rostrup E., Sørndergaard L., Ring P.B., Amtorp O., Larsson H.B.W. (1998). Capillary transfer constant of Gd-DTPA in the myocardium at rest and during vasodilation assessed by MRI. Magn. Reson. Med..

[20] Li K.L., Lewis D., Coope D.J., Roncaroli F., Agushi E., Pathmanaban O.N., King A.T., Zhao S., Jackson A., Cootes T. (2021). The LEGATOS technique: A new tissue-validated dynamic contrast-enhanced MRI method for whole-brain, high-spatial resolution parametric mapping. Magn. Reson. Med..

[21] Lewis D., McHugh D.J., Li K.-L., Zhu X., Mcbain C., Lloyd S.K., Jackson A., Pathmanaban O.N., King A.T., Coope D.J. (2021). Detection of early changes in the post-radiosurgery vestibular schwannoma microenvironment using multinuclear MRI. Sci. Rep..

[22] Tofts P.S., Brix G., Buckley D.L., Evelhoch J.L., Henderson E., Knopp M.V., Larsson H.B., Lee T.-Y., Mayr N.A., Parker G.J. (1999). Estimating kinetic parameters from dynamic contrast-enhanced t1-weighted MRI of a diffusable tracer: Standardized quantities and symbols. J. Magn. Reson. Imaging.

[23] Sasi S.D., Gupta R.K., Patir R., Ahlawat S., Vaishya S., Singh A. (2021). A comprehensive evaluation and impact of normalization of generalized tracer kinetic model parameters to characterize blood-brain-barrier permeability in normal-appearing and tumor tissue regions of patients with glioma. Magn. Reson. Imaging.

[24] Li K.L., Zhu X., Zhao S., Jackson A. (2017). Blood-brain barrier permeability of normal-appearing white matter in patients with vestibular schwannoma: A new hybrid approach for analysis of T1 -W DCE-MRI. J. Magn. Reson. Imaging.

[25] Sengupta A., Agarwal S., Gupta P.K., Ahlawat S., Patir R., Gupta R.K., Singh A. (2018). On differentiation between vasogenic edema and non-enhancing tumor in high-grade glioma patients using a support vector machine classifier based upon pre and post-surgery MRI images. Eur. J. Radiol..

[26] Li K.-L., Lewis D., Jackson A., Zhao S., Zhu X. (2018). Low-dose T1W DCE-MRI for early time points perfusion measurement in patients with intracranial tumors: A pilot study applying the microsphere model to measure absolute cerebral blood flow. J. Magn. Reson. Imaging.

[27] Larsson H.B., Hansen A.E., Berg H.K., Rostrup E., Haraldseth O. (2008). Dynamic contrast-enhanced quantitative perfusion measurement of the brain usingT1-weighted MRI at 3T. J. Magn. Reson. Imaging.

[28] Jackson A. (2003). Imaging microvascular structure with contrast enhanced MRI. Br. J. Radiol..

[29] Evans D.R. (2009). Neurofibromatosis type 2 (NF2): A clinical and molecular review. Orphanet J. Rare Dis..

[30] Evans D.G., Howard E., Giblin C., Clancy T., Spencer H., Huson S.M., Lalloo F. (2010). Birth incidence and prevalence of tumor-prone syndromes: Estimates from a UK family genetic register service. Am. J. Med. Genet. A.

[31] Stivaros S.M., Stemmer-Rachamimov A.O., Alston R., Plotkin S.R., Nadol J.B., Quesnel A., O’Malley J., Whitfield G.A., McCabe M.G., Freeman S.R. (2015). Multiple synchronous sites of origin of vestibular schwannomas in neurofibromatosis Type 2. J. Med. Genet..

[32] Lloyd S.K., Evans D.G. (2013). Neurofibromatosis type 2 (NF2): Diagnosis and management. Handb. Clin. Neurol..

[33] Evans D.G., Stivaros S.M. (2014). Multifocality in neurofibromatosis type 2. Neuro-Oncology.

[34] Hannan C.J., Hammerbeck-Ward C., Pathmanaban O.N., Smith M.J., Rutherford S.A., Lloyd S.K., Freeman S.R.M., Wallace A.J., King A.T., Evans D.G.R. (2022). Multiple Meningiomas as a Criterion for the Diagnosis of Neurofibromatosis Type 2 and Other Tumor Predisposition Syndromes. Neurosurgery.

[35] Plotkin S.R., Merker V.L., Halpin C., Jennings D., McKenna M.J., Harris G.J., Barker F.G. (2012). Bevacizumab for progressive vestibular schwannoma in neurofibromatosis type 2: A retrospective review of 31 patients. Otol. Neurotol..

[36] Mautner V.-F., Nguyen R., Kutta H., Fuensterer C., Bokemeyer C., Hagel C., Friedrich R.E., Panse J. (2010). Bevacizumab induces regression of vestibular schwannomas in patients with neurofibromatosis type 2. Neuro-oncology.

[37] Plotkin S.R., Stemmer-Rachamimov A.O., Barker F.G., Halpin C., Padera T.P., Tyrrell A., Sorensen A.G., Jain R.K., di Tomaso E. (2009). Hearing improvement after bevacizumab in patients with neurofibromatosis type 2. N. Engl. J. Med..

[38] Sourbron S.P., Buckley D.L. (2011). On the scope and interpretation of the Tofts models for DCE-MRI. Magn. Reson. Med..

[39] Chang Y.-C.C., Ackerstaff E., Tschudi Y., Jimenez B., Foltz W., Fisher C., Lilge L., Cho H., Carlin S., Gillies R.J. (2017). Delineation of Tumor Habitats based on Dynamic Contrast Enhanced MRI. Sci. Rep..

[40] Patlak C.S., Blasberg R.G. (1985). Graphical evaluation of blood-to-brain transfer constants from multiple-time uptake data. Generalizations. J. Cereb. Blood Flow Metab..

[41] Ewing J.R., Knight R.A., Nagaraja T.N., Yee J.S., Nagesh V., Whitton P.A., Li L., Fenstermacher J.D. (2003). Patlak plots of Gd-DTPA MRI data yield blood-brain transfer constants concordant with those of14C-sucrose in areas of blood-brain opening. Magn. Reson. Med..

[42] Durukan A., Marinkovic I., Strbian D., Pitkonen M., Pedrono E., Soinne L., Abo-Ramadan U., Tatlisumak T. (2009). Post-ischemic blood–brain barrier leakage in rats: One-week follow-up by MRI. Brain Res..

[43] Abo-Ramadan U., Durukan A., Pitkonen M., Marinkovic I., Tatlisumak E., Pedrono E., Soinne L., Strbian D., Tatlisumak T. (2009). Post-ischemic leakiness of the blood–brain barrier: A quantitative and systematic assessment by Patlak plots. Exp. Neurol..

[44] Taheri S., Gasparovic C., Shah N.J., Rosenberg G.A. (2011). Quantitative measurement of blood-brain barrier permeability in human using dynamic contrast-enhanced MRI with fast T1 mapping. Magn. Reson. Med..

[45] Chang L.C., Koay C.G., Basser P.J., Pierpaoli C. (2008). Linear least-squares method for unbiased estimation of T1 from SPGR signals. Magn. Reson. Med..

[46] Li K.L., Zhu X.P., Waterton J., Jackson A. (2000). Improved 3D quantitative mapping of blood volume and endothelial permeability in brain tumors. J. Magn. Reson. Imaging.

[47] Li K.L., Zhu X.P., Checkley D.R., Tessier J.J.L., Hillier V.F., Waterton J.C., Jackson A. (2003). Simultaneous mapping of blood volume and endothelial permeability surface area product in gliomas using iterative analysis of first-pass dynamic contrast enhanced MRI data. Br. J. Radiol..

[48] Batchelor T.T., Sorensen A.G., di Tomaso E., Zhang W.-T., Duda D.G., Cohen K.S., Kozak K.R., Cahill D.P., Chen P.-J., Zhu M. (2007). AZD2171, a Pan-VEGF Receptor Tyrosine Kinase Inhibitor, Normalizes Tumor Vasculature and Alleviates Edema in Glioblastoma Patients. Cancer Cell.

[49] Pishko G.L., Muldoon L.L., Pagel M.A., Schwartz D.L., Neuwelt E.A. (2015). Vascular endothelial growth factor blockade alters magnetic resonance imaging biomarkers of vascular function and decreases barrier permeability in a rat model of lung cancer brain metastasis. Fluids Barriers CNS.

[50] Gerstner E.R., Emblem K.E., Chang K., Vakulenko-Lagun B., Yen Y.F., Beers A.L., Dietrich J., Plotkin S.R., Catana C., Hooker J.M. (2020). Bevacizumab Reduces Permeability and Concurrent Temozolomide Delivery in a Subset of Patients with Recurrent Glioblastoma. Clin. Cancer Res..

[51] Wong H.K., Lahdenranta J., Kamoun W.S., Chan A.W., McClatchey A.I., Plotkin S.R., Jain R.K., di Tomaso E. (2010). Anti–Vascular Endothelial Growth Factor Therapies as a Novel Therapeutic Approach to Treating Neurofibromatosis-Related Tumors. Cancer Res..

[52] Navis A.C., Hamans B.C., Claes A., Heerschap A., Jeuken J.W., Wesseling P., Leenders W.P. (2011). Effects of targeting the VEGF and PDGF pathways in diffuse orthotopic glioma models. J. Pathol..

[53] Buemi F., Guzzardi G., Del Sette B., Sponghini A.P., Matheoud R., Soligo E., Trisoglio A., Carriero A., Stecco A. (2019). Apparent diffusion coefficient and tumor volume measurements help stratify progression-free survival of bevacizumab-treated patients with recurrent glioblastoma multiforme. Neuroradiol. J..

[54] Cazzador D., Astolfi L., Daloiso A., Tealdo G., Simoni E., Mazzoni A., Zanoletti E., Marioni G. (2023). Tumor Microenvironment in Sporadic Vestibular Schwannoma: A Systematic, Narrative Review. Int. J. Mol. Sci..

[55] Varatharaj A., Liljeroth M., Darekar A., Larsson H.B., Galea I., Cramer S.P. (2018). Blood-brain barrier permeability measured using dynamic contrast-enhanced magnetic resonance imaging: A validation study. J. Physiol..

[56] Heye A.K., Thrippleton M.J., Armitage P.A., Hernández M.d.C.V., Makin S.D., Glatz A., Sakka E., Wardlaw J.M. (2016). Tracer kinetic modelling for DCE-MRI quantification of subtle blood–brain barrier permeability. Neuroimage.

[57] Deng X., Liu L., Luo J., Liu L., Hui X., Feng H. (2022). Research on the Mechanism of Cognitive Decline in Patients with Acoustic Neuroma. Front. Neurosci..

[58] Deng X., Liu L., Zhen Z., Chen Q., Liu L., Hui X. (2022). Cognitive decline in acoustic neuroma patients: An investigation based on resting-state functional magnetic resonance imaging and voxel-based morphometry. Front. Psychiatry.

[59] Deng X., Liu L., Li J., Yao H., He S., Guo Z., Sun J., Liu W., Hui X. (2022). Brain structural network to investigate the mechanism of cognitive impairment in patients with acoustic neuroma. Front. Aging Neurosci..

[60] Wang X., Xu P., Li P., Wang Z., Zhao F., Gao Z., Xu L., Luo Y.-J., Fan J., Liu P. (2016). Alterations in gray matter volume due to unilateral hearing loss. Sci. Rep..

[61] Kurtcan S., Alkan A., Kilicarslan R., Bakan A.A., Toprak H., Aralasmak A., Aksoy F., Kocer A. (2016). Auditory Pathway Features Determined by DTI in Subjects with Unilateral Acoustic Neuroma. Clin. Neuroradiol..

[62] Li K.-L., Buonaccorsi G., Thompson G., Cain J.R., Watkins A., Russell D., Qureshi S., Evans D.G., Lloyd S.K., Zhu X. (2012). An improved coverage and spatial resolution-using dual injection dynamic contrast-enhanced (ICE-DICE) MRI: A novel dynamic contrast-enhanced technique for cerebral tumors. Magn. Reson. Med..

[63] Koenig M., Klotz E., Luka B., Venderink D.J., Spittler J.F., Heuser L. (1998). Perfusion CT of the brain: Diagnostic approach for early detection of ischemic stroke. Radiology.

[64] Keil V.C., Mädler B., Gieseke J., Fimmers R., Hattingen E., Schild H.H., Hadizadeh D.R. (2017). Effects of arterial input function selection on kinetic parameters in brain dynamic contrast-enhanced MRI. Magn. Reson. Imaging.

[65] Lewis D., Zhu X., Coope D.J., Zhao S., King A.T., Cootes T., Jackson A., Li K.-L. (2022). Surrogate vascular input function measurements from the superior sagittal sinus are repeatable and provide tissue-validated kinetic parameters in brain DCE-MRI. Sci. Rep..

[66] Ashburner J., Friston K. (1997). Multimodal Image Coregistration and Partitioning—A Unified Framework. NeuroImage.

